# Rapture of renal angiomyolipoma during pregnancy: a case report

**DOI:** 10.1186/1757-1626-1-245

**Published:** 2008-10-17

**Authors:** Stylianos Kontos, Vasilios Politis, Ioannis Fokitis, Georgios Lefakis, Georgios Koritsiadis, Vasileios Simaioforidis, Stefanos Kachrilas, Evangelos Chatziplis, Sotirios Koritsiadis

**Affiliations:** 1 Department of Urology, General Hospital of Nikea, 3 D. Mantouvalou St., Nikea, 18454, Piraeus, Greece; 2 First Department of Urology, Laiko General Hospital, University of Athens, School of Medicine, Athens, Greece

## Abstract

**Introduction:**

Rapture of a renal angiomyolipoma and massive retroperitoneal hemorrhage, during pregnancy is rare and occasionally fatal. The association of this complication with pregnancy has been reported sporadically in the literature.

**Case presentation:**

We report a case of a 28 years old woman, in 33 week of her first pregnancy who came to our hospital complaining of abdominal pain in the right hemiabdomen, associated with nausea and vomiting. The ultrasound and MRI (Magnetic resonance imaging) scan showed a 7 × 7 × 5 cm mass suggestive of angiomyolipoma in the right kidney, with evidence of retroperitoneal bleeding right perirenal and intrarenal haematoma. Given the size of the tumor, presence of symptoms and hemodynamic instability of the patient right nephrectomy was performed, following emergency caesarean delivery. The histological study of the resected mass revealed the presence of angiomyolipoma.

**Conclusion:**

In conclusion, it seems that these tumours show a greater growth index in pregnant women. Angiomyolipoma with spontaneous bleeding during pregnancy is a dangerous condition that may cause mortality in the mother and fetus, but elective, simultaneous cesarean section and radical nephrectomy can be performed. We have also done a review of the literature focusing on its management and its relationship with pregnancy.

## Introduction

Renal angiomyolipoma (AML) is a relatively infrequent clinical entity observed in 0.3% of the general population and accounting for 3% of all solid renal masses. AMLs are benign mesothelial tumors, with three histologic characteristics: mature adipose tissue, blood vessels, and smooth muscle cells. Most of AMLs are asymptomatic and found incidentally on imaging examinations. It has been described 2 types:isolated angiomyolipoma and angiomyolipoma associated with sclerosis. When AML is associated with complex clinical situations, such as developed in a solitary kidney, bilateral presence, large or multicentric tumors of those associated with tuberous sclerosis (Bourneville's disease), or pregnancy, have medical interest and the management of these are quite challenging. To the best of our knowledge, during the past 10 years only three cases of massive retroperitoneal hemorrhage, resulting from rupture of a renal angiomyolipoma during pregnancy have occurred. In addition of report of this rare medical case we include brief comments of pathophysiology, diagnosis and treatment of this rare tumor.

## Case presentation

We report a case of a 28 years old woman, in 33 week of her first pregnancy, who was admitted to our department for severe right flank pain, detected in right hypochondrium, associated with nausea, vomiting, and irritative bladder symptoms. Personal and familial histories were unremarkable. The patient was hemodynamically stable without hematuria, lumbar pain or other urological symptoms. Physical examination revealed no specific findings, a good general condition, an axillary temperature of 38°C, blood pressure of 120/75 mmHg and a heart rate of 78 bpm. Abdominal palpation revealed no masses. The only pathological laboratory test parameter was the hemoglobin 8,7 g/dl and hematocrit of 25,5%, that required the transfusion of two red cell concentrate units.

**Figure 1:**
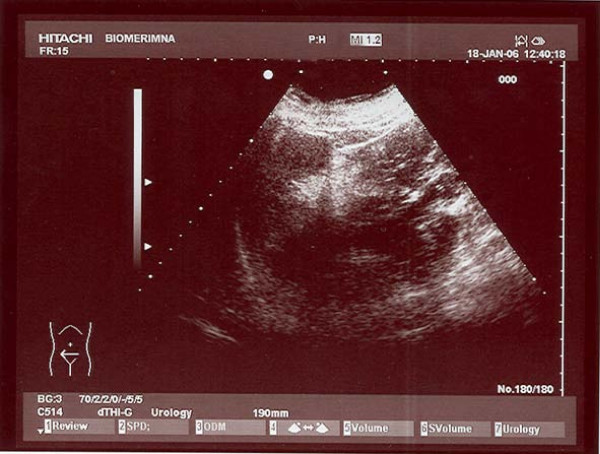
Renal ultrasonogram depicting an echogenic mass in the right kidney the inferior aspect of the kidney that anteriorly displaces the renal sinus.

**Figure 2:**
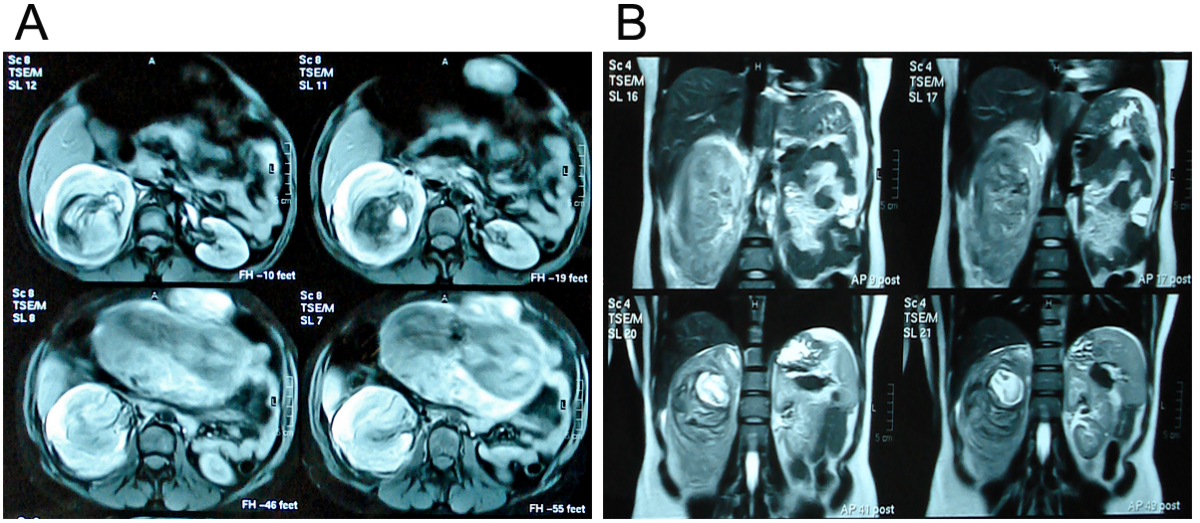
**(A):Abdominal MRI view showing the presence of blood accumulation in the right perirenal space, with destructuring of the renal architecture and the presence of a right intrarenal hematoma.** (B): Coronal abdominal MRI view showing the presence of right renal masse, with signs of bleeding.

Abdominal ultrasound examination revealed a mass, with mixed echogenity, without acoustic shadowing well circumscribed, expanding at the upper pole of right kidney (Fig 1). The mass confirmed with MRI, measuring approximately 7 × 7 × 5 cm in size with evidence of recent extensive retroperitoneal bleeding, with right perirenal and intrarenal hematoma.(Fig 2a–b.).

After a couple of hours she was developed an episode of fetal bradycardia, hypotension, and a hematocrit continued to decline, despite repeated blood transfusion, which combined with symptoms of intense lumbar pain and hematuria.

**Figure 3:**
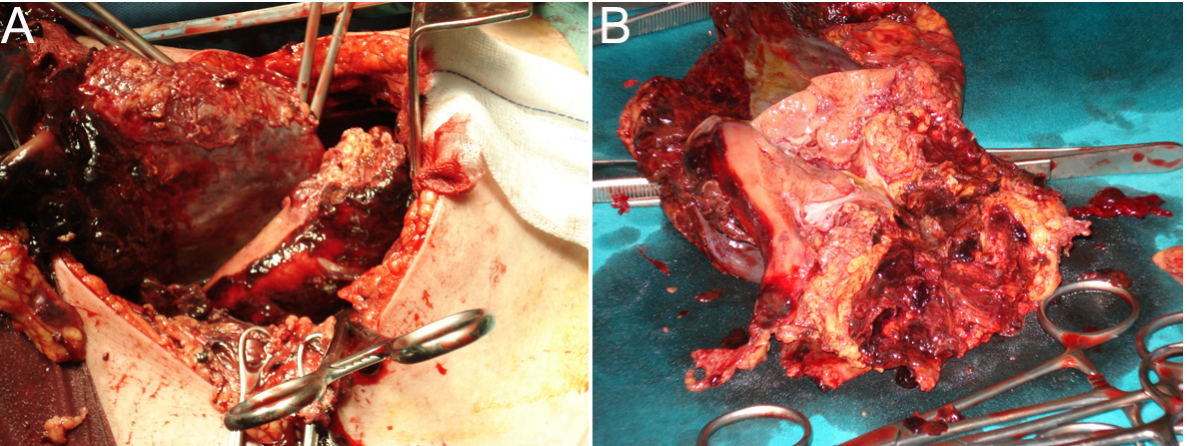
(A and B): Photograph of the fresh cut specimen shows a tan angiomyolipoma hemorrhagic angiomyolipoma.

Considering the hemodynamic instability of the patient, emergency cesarean delivery, under general anaesthesia, was undertaken, because of foetal distress. Exploration of the retroperitoneal space after foetal extraction, confirmed the presence of a large haematoma and the renal mass., which occupied the intrarenal space (Fig. 3a). Right nephrectomy was performed, and the haemorrhaging contents was evacuated. (Fig 3b.).

**Figure 4:**
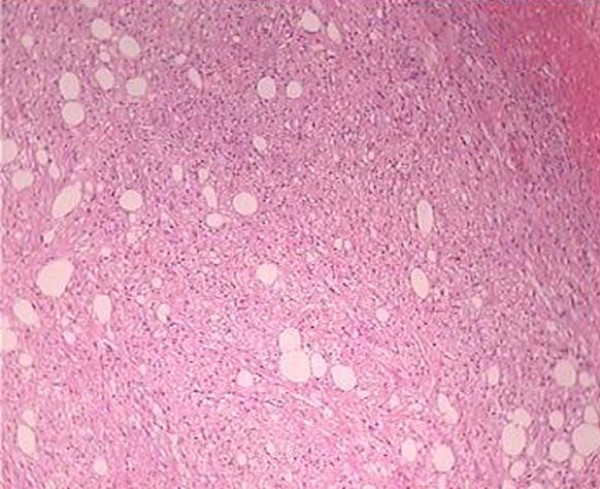
Photomicrograph (original magnification, x40; hematoxylin-eosin stain) of one of the numerous tan nodules that replaced the renal parenchyma shows smooth muscle thick-walled blood vessels and fatty tissue.

The histological study of the resected mass revealed the presence of with admixture of mature adipose tissue, smooth muscle, and thick-walled blood vessels.(Fig. 4)

A healthy male infant was delivered and the patient had an uneventful recovery.

## Discussion

Renal AML are not very frequent in urologic activities, especially when it is associated with pregnancy. The majority of AMLs are asymptomatic [1], but the increasingly widespread application of ultrasound, CT (computer tomography) and MRI (Magnetic resonance imaging) are diagnostic exploratory techniques of the renal mass [2]. In our patient the finding of the renal tumor mass, during the symptomatic phase when the diaforodiagnostic problem occurred, was the result of an ultrasound examination.

The majority of this kind of tumor, are often solitary, the mean age of presentation is 43 years, 4 times more common in men and, interestingly, involve the right kidney [3] Palpable abdominal mass, hematuria or flank pain are the main symptoms, and acute abdominal or even shock are the results of spontaneous rupture of the tumor [4]. The histopathological pattern of the AML consists of mature adipose tissue, containing numerous blood vessel structures within smooth muscles layers and accumulations, histiocytes, macrophages and lymphocytes [5].

The true nature of the tumor has not been fully elucidated. Immunological mechanisms seems to be related, since increased numbers of estrogen and progesterone receptors at muscle cell level and the tumour cells contain melanosomes and synthesize melanin because they contain dopa oxidase, are helpful microscopical findings to determine the best management approach [6,7]. This hypothesis may be supported by the hormonal profile of pregnant women, due to the presence of progesterone receptors.

The management of AML is widely discussed in the literature. Asymptomatic tumors smaller than 4 cm in size should be subjected to periodic ultrasound and CT controls every 6 months [8,9]. Symptomatic, bilateral lesions should be treated with selective arterial embolization or partial nephrectomy. Radical nephrectomy is required when the patient is hemodynamic unstable, due to retroperitoneal hemorrhage [10]. In our patient, the life threatening hemodynamic profile, in combination with fetal pulse abnormality, required emergency caesarian section and at the same time control of retroperitoneum bleeding, with radical right nephrectomy.

In conclusion, it seems that these tumors show a greater growth index in pregnant women and the question that may be raised is when is the appropriate time for surgical interference. The second trimester of pregnancy seems to be ideal [11], since the risk of fetal organogenetic abnormalities decreases, even though the need of individualization of each case is necessary.

## Abbreviations

AML: angiomyolipoma; MRI: Magnetic resonance imaging; CT: computer tomography.

## Consent

Written informed consent was obtained from the patient for publication of this case report and accompanying images. A copy of the written consent is available for review by the Editor-in Chief of this journal.

## Competing interests

The authors declare that they have no competing interests.

## Authors' contributions

All authors have made substantial contribution to concept this case reports
